# Changes in renal WT-1 expression preceding hypertension development

**DOI:** 10.1186/s12882-016-0250-6

**Published:** 2016-03-24

**Authors:** Luciana Mazzei, Mercedes García, Juan Pablo Calvo, Mariana Casarotto, Miguel Fornés, María Angélica Abud, Darío Cuello-carrión, León Ferder, Walter Manucha

**Affiliations:** National Scientific and Technical Research Council, Institute of Medical and Experimental Biology of Cuyo, Mendoza, Argentina; Pathology Department, Pharmacology Area Medical Sciences College, National University of Cuyo, Mendoza, CP5500 Argentina; National Scientific and Technical Research Council, Institute of Histology and Embryology of Mendoza, Mendoza, Argentina; Department of Physiology and Pharmacology, School of Medicine, Puerto Rico University, Puerto Rico, EEUU USA

**Keywords:** Hypertension, Nephrogenesis, Wilms’ tumor 1 factors, Heat shock protein 70, Vitamin D receptor, Mitochondria

## Abstract

**Background:**

Hypertension is a public health problem with mostly unknown causes, and where strong hereditary genetic alterations have not been fully elucidated. However, the use of experimental models has provided valuable information. Recent evidences suggest that alterations in key nephrogenic factors, such as Wilms’ tumor 1 transcription factor (WT-1), could contribute to the development of hypertension. The aim of this paper is to evaluate the expression of WT-1 and related genes in the nephrogenic process in connection with the development of hypertension as well as the corresponding anatomical and functional correlation.

**Methods:**

Male spontaneously hypertensive and control rats were evaluated weekly from birth until week 8 of life. Their blood pressure was taken weekly using the tail-cuff blood pressure system. Weekly, 5 rats per group were sacrificed with a lethal injection of pentobarbital, and their kidneys were removed, decapsulated and weighed. The serum was collected for measuring biochemical parameters. The results were assessed using one-way analysis of variance for comparisons between groups.

**Results:**

The relationship between renal weight/total body weights was established, without significantly different values. These data were compared with apoptosis, fibrosis, number and size of the glomeruli. The elevation of systolic blood pressure was significant since week 6. Biochemical values differed slightly. Histology showed a slight increase in deposits of collagen fibers since week 4. Additionally, in kidney cortices, the expression of WT-1, heat shock protein 70 (Hsp70) and vitamin D receptors (VDR) decreased since week 4. Finally, we demonstrated ultrastructural damage to mitochondria since week 4.

**Conclusions:**

Our results would suggest an unprecedented link, possibly a regulatory mechanism, between WT-1 on nephrogenic alteration processes and their relationship with hypertension. Moreover, and previous to the increase in blood pressure, we demonstrated low expressions of WT-1, VDR and Hsp70 in kidneys from neonatal SHRs. If so, this may suggest that deregulation in the expression of WT-1 and its impact on nephrogenesis induction could be crucial in understanding the development and maintenance of hypertension.

**Electronic supplementary material:**

The online version of this article (doi:10.1186/s12882-016-0250-6) contains supplementary material, which is available to authorized users.

## Background

Hypertension is one of the most complex diseases to understand due to its strong genetic component. Although the genetic basis of the disease is firmly established and there have been very important developments in the field of molecular biology in recent years, few studies deal with hypertension as a result of possible changes in renal development by decoupling organogenic key factors [1]. In this regard, nephrogenic impairment is often associated with low birth weight and has been recognized as a powerful risk factor for kidney disease with low glomerular filtration rate, which would condition a more rapid progression of kidney disease and hypertension [2]. Of special interest is the fact that one important consequence of the altered nephrogenic process is hypertension. Kidney damage is a significant event in the development of hypertension and, thus, maternal food restriction alters key fetal nephrogenesis gene expression, such as Wilms’ tumor transcription factor 1 (WT-1) and other factors; this appears to contribute to hypertension development [3, 4]. In addition, some genes involved in hypertension development also depend on WT-1. The fact that renin gene transcription is regulated by WT-1, and that inherited mutations in the WT-1 gene can lead to hypertension, may explain the findings of patients with high renin and hypertension [5]. Accordingly, unpublished results from our laboratory assays performed in adult spontaneously hypertensive rats (SHRs) suggested an opposite relationship between WT-1 renal expression and blood pressure values. Furthermore, recent hypertension studies show low levels of vitamin D associated with the exaltation of the renin-angiotensin-aldosterone system (RAAS) [6–8]; and, in that respect, our laboratory has demonstrated, in the primary culture of proximal tubular cells of SHRs, that heat shock protein 70 (Hsp70) protects against hypertension induced by angiotensin II by exerting a cytoprotective effect [9]. We have also demonstrated in adult SHRs that the induction of vitamin D receptor (VDR) modulates an increase in Hsp70 levels, with a decrease in the angiotensin II receptor, type 1 (AT_1_) expression, providing renal protection [10]. These contributions are relevant considering previous reports, where WT-1 and Hsp70 are physically associated (localized) in embryonic rat kidney cells, in primary Wilms’ tumor samples, and in cultured cells with inducible WT-1 expression [11]. It has also been described that Hsp70 is an important cofactor for the function of WT-1, and suggested a potential role for this chaperone during nephrogenesis. Furthermore, it has been reported that human VDR is regulated by WT-1 [12]. These findings strengthen our recent demonstrations in the adult SHR model. Here we established, in mitochondria, a significant and consistent anatomical-functional correlate with the expression of the markers/modulators mentioned above (AT_1_, VDR and Hsp70) [10]. Moreover, cumulative evidence suggests that reactive oxidative stress (ROS) and inflammation, with particular attention to mitochondria, plays an important role in hypertension development [13, 14]. In this regard, our laboratory reviewed and proposed a complex new regulation in the inflammatory pathway that involves the possible modulation of Hsp70 and WT-1 on mitochondrial signaling and where the net effect would favor cell survival by WT-1 stabilizing Bcl_2_, and would limit the potential for the release of cytochrome C from mitochondria [15].

Finally, SHRs were originally inbred from Wistar rats and their Wistar–Kyoto (WKY) inbred non-hypertensive controls. These rats develop hypertension at about 6 weeks of age without physiological, pharmacological or surgical intervention; however, environmental factors affect the development of hypertension, and the importance of this model has been attributed to the similarity of its pathophysiology with essential hypertension in humans [16].

Based on the arguments stated above, we finally proposed to evaluate the following key hypothesis: Changes in the expression pattern of the WT-1 transcription factor and molecular mediators related to nephrogenesis could contribute to anatomical and functional kidney disorders, as well as to a hemodynamics disorders typical of the SHR model. Such modifications could respond to a Hsp70-dependent conditioning, as well as a probable modulation of the vitamin D receptor. In addition, we discuss the possibility that mitochondrial injury and dysfunction linked to master nephrogenic factors, such as WT-1, could play a central role in the pathogenesis of the essential hypertension model.

## Methods

Both the SHR group and the control group, formed by Wistar Kyoto rats (WKY), were cared in accordance with the Guiding Principles in the Care and Use of Animals of the United States National Institute of Health. All experimental procedures were previously approved by the Institutional Animal Care and Use Committee of the Medical Sciences College, National University of Cuyo (Protocol approval N° 46/2015).

### Animals

Newborn male SHRs (*n* = 45) and WKYs (*n* = 45), were evaluated during their first 8 weeks of life (from birth, i.e. week 0, to week 8). Their blood pressure was taken weekly using the CODA tail-cuff blood pressure system (Kent Scientific Corporation). Weekly, 5 rats per group (SHR and WKY), were sacrificed with a lethal injection of pentobarbital, and their kidneys were removed, decapsulated and weighed. The serum was collected for measuring biochemical parameters. The serum urea and creatinine concentrations were determined using colorimetric assays (Sigma kits).

### Histological studies

Sections of the renal cortex were processed for histological studies according to previously described [17].

### Morphometric evaluation of interstitial fibrosis

For all morphologic evaluations, the observer was blinded to the origin of the histological sections. A standard point counting method was used to quantitate the fibrosis of the renal interstitium [18–20]. Results were expressed as percentage of measured area.

### Glomerular morphometric analysis

Masson’s trichrome stained sagittal sections of the rat kidney cortices were scanned under light microscopy with a zig-zag movement so as to assure equal sampling of the cortex and to avoid evaluating the same glomerulus twice. The morphometric parameters were evaluated as previously [19], with minor modifications. The relative number of glomeruli per square millimeter of renal cortex was calculated. A reticulate eyepiece was used for counting glomeruli. To avoid double counting, glomeruli crossed by the upper and left margins of the reticle were counted in 10 fields of tissue section. The number of glomeruli per microscopic field was counted in every section observed (100 fields). To assess the relative glomerular diameter, a micrometric eyepiece was used. Ten tissue sections per microscopic field were taken at random. The diameters were expressed in micrometers (μm).

### Identification of cellular apoptosis: the TUNEL technique

Cellular apoptosis by terminal deoxynucleotidyl transferase-mediated dUTP nick end labeling technique was performed according to previously reported [20].

### Electron microscopy

The tissue samples were processed for electron microscopy according to previously described methodology with minor modifications [20].

### Immunohistochemical studies

Kidney paraffin sections were processed according to previously described technique with minor modifications [21]. The antibodies applied were rabbit polyclonal antibody against WT-1 (C-19), mouse monoclonal antibody against VDR (D-6), rabbit polyclonal antibody against Hsp70 (H-300) and rabbit polyclonal antibody against AT_1_ (306) (Santa Cruz Biotechnology, Inc.), diluted at 1:500. A commercial immunoperoxidase kit was used (Dako). The positive reaction was evaluated considering the specific location of the immunostaining and the intensity of the immunoreaction. The negative controls included tissues unexposed to primary antibodies as well as tissues exposed to control immunoglobulin G. The positive controls were human breast cancer biopsy samples. The immunostaining was evaluated and resolved by consensus according to a scoring system reported previously [22, 23].

### Immunofluorescence confocal microscopy

The kidneys were processed according to previously described technique [21]. Primary antibodies were: polyclonal anti-WT-1 (1:100) (Santa Cruz Biotechnology), monoclonal anti-Hsp70 (1:100) (Sigma Aldrich), monoclonal anti-VDR (1:100) (Santa Cruz Biotechnology), and polyclonal anti-AT_1_ (1:100) (Santa Cruz Biotechnology). Secondary antibodies were: goat anti-rabbit IgG antibody, Cy2 conjugated (1:750) and donkey anti-mouse IgG antibody, Cy3 conjugated (1:750). After being washed, the tissues were stained with Hoechst 33342 (10 nM) for 5 min. The coverslips were mounted in Fluoroshield solution (Sigma Aldrich) for confocal microscopy. Confocal images were taken using FV10-ASW 1.7 (Olympus IX81 microscope).

### Mitochondria isolation from tissue

Mitochondria isolation and purity of mitochondrial fractions were established as previously described [24, 25] with minor modifications.

### NADPH activity assay

NADPH oxidase activity was measured in enriched mitochondrial fractions of the renal cortex using the Luminol (5-amino-2, 3-dihydro-1, 4-phthalazine; Sigma-Aldrich) technique. The values were expressed as relative fluorescence units (RFU) per microgram of protein and per minute of incubation.

### Statistical analysis

The results were assessed using one-way analysis of variance (ANOVA) for comparisons between groups. Differences between groups were determined using the Bonferroni posttest. A *p* < 0.05 was considered to be significant. Results are given as mean ± standard error of the mean (SEM). Statistical tests were performed using GraphPad InStat version 3.00 for Windows 95 (GraphPad Software, Inc., La Jolla, California, United States of the America).

## Results

### Weight, hemodynamics and serum chemistry

The body weights in each strain increased rapidly from newborn to 8 weeks of life. However, the differences in body weights between the two strains were enlarged after week 6, and by week 8 the SHRs and WKYs weighed 160 ± 10 g and 190 ± 12 g (*p* < 0.05), respectively (*n* = 5 per week/per group). We also evaluated the renal weight/total body weight ratio, and found no significant differences between the strains (SHR vs. WKY; *p* = NS) in the weeks studied (*n* = 5 per week/per group). On the other hand, due to the low weight of the animals, we could only measure the systolic blood pressure (SBP) accurately since week 4 of life until the end of the study (week 8). The SBP in both strains, SHR and WKY, was 195 ± 10 vs. 145 ± 8 mmHg; *p* < 0.05 at week 6. Thereafter, the blood pressure of the SHRs elevated to 250 mmHg at week 8. In contrast, the SBP of the WKYs was 145 ± 8 and 150 ± 10 mmHg at weeks 6 and 8 (*p* = NS) (*n* = 5 per week/per group) (Table 1).

**Table 1:** Body weight, kidney/body weight ratio, and blood pressure in SHRs and WKYs

Strain	0-week-old rat	1-week-old rat	2-week-old rat	3-week-old rat	4-week-old rat	5-week-old rat	6-week-old rat	7-week-old rat	8-week-old rat
SHR									
Body weight (g)	6 ± 1	13 ± 3	24 ± 4	45 ± 6	82 ± 7	100 ± 10	130 ± 9*	150 ± 9*	160 ± 10*
WKY									
Body weight (g)	7 ± 1	17 ± 4	29 ± 5	54 ± 7	92 ± 8	115 ± 9	150 ± 8	175 ± 10	190 ± 12
SHR									
Renal/Body weight (mg/g)	5.60 ± 0.3	6.05 ± 0.90	5.9 ± 1.00	5.86 ± 0.90	6.02 ± 1.10	7 ± 1.30	7.7 ± 1.00	7.5 ± 1.20	8 ± 1.20
WKY									
Renal/Body weight (mg/g)	6.1 ± 0.30	7.43 ± 1.10	6.66 ± 1.10	6.9 ± 1.20	6.82 ± 1.20	6.3 ± 1.20	7.3 ± 1.10	6.7 ± 1.10	7.3 ± 1.10
SHR									
SBP (mmHg)	-------	-------	-------	-------	142 ± 11	150 ± 10	195 ± 10**	230 ± 15**	250 ± 20**
WKY									
SBP (mmHg)	-------	-------	-------	-------	135 ± 8	140 ± 7	145 ± 8	148 ± 7	150 ± 10

Significantly different from the values at 6, 7 and 8 weeks of age in each strain (*p < 0.05 and **p < 0.01). Results are mean ± SEM; *n* = 5

The study of biochemical parameters (*n* = 5 per week/per group) showed an increasing tendency for both serum urea and creatinine of hypertensive rats (SHR) and their normotensive controls (WKY). However, since week 4, creatinine values in SHRs were slightly lower than in WKYs (0.65 ± 0.03 vs. 0.75 ± 0.04 mg/dl; *p* = NS). In contrast, the values of plasma urea in SHRs were discreetly higher than in WKYs (15 ± 2 vs. 13 ± 1.6 mg/dl; *p* = NS). Relevant to our study was that, at week 8, creatinine values in SHRs were lower than in WKYs (0.70 ± 0.06 vs. 0.95 ± 0.07 mg/dl; *p* < 0.05), while the values of plasma urea in SHRs were higher than in WKYs (20 ± 2 vs. 15 ± 2 mg/dl; *p* < 0.05) (Table 2).

**Table 2:** Biochemical parameters in SHRs and WKYs

Strain	0-week-old rat	1-week-old rat	2-week-old rat	3-week-old rat	4-week-old rat	5-week-old rat	6-week-old rat	7-week-old rat	8-week-old rat
SHR									
Creatinine									
(mg/dl)	0.49 ± 0.08	0.51 ± 0.07	0.55 ± 0.06	0.60 ± 0.05	0.65 ± 0.03	0.67 ± 0.09	0.68 ± 1.00	0.70 ± 0.09	0.70 ± 0.06*
WKY									
Creatinine									
(mg/dl)	0.55 ± 0.07	0.60 ± 0.08	0.65 ± 0.06	0.70 ± 0.07	0.75 ± 0.04	0.79 ± 0.09	0.85 ± 0.09	0.90 ± 1.10	0.95 ± 0.07
SHR									
Urea									
(mg/dl)	12 ± 1.00	13 ± 1.00	14 ± 2.00	15 ± 2.00	15 ± 2.00	16 ± 2.00	18 ± 3.00	20 ± 3.00	20 ± 2.00*
WKY									
Urea									
(mg/dl)	11 ± 1.00	12 ± 2.00	12 ± 2.00	13 ± 2.00	13 ± 1.60	14 ± 2.00	14 ± 2.00	15 ± 4.00	15 ± 2.00

Significantly different from the values at 8 weeks of age in each strain (*p < 0.05). Results are mean ± SEM; *n* = 5

### Interstitial fibrosis, apoptosis, and ultrastructural mitochondrial damage during hypertension development

**Fig. 1:**
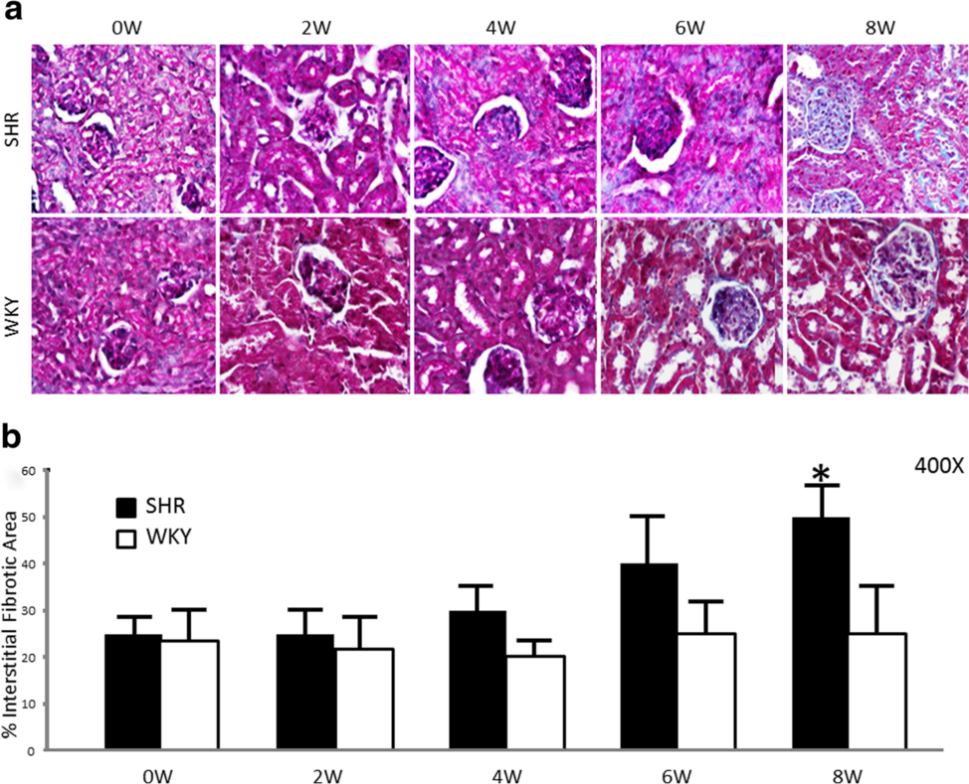
Morphometric evaluation of interstitial fibrosis. **a** Masson’s trichrome-stained sections of kidney cortices from neonatal rats (SHRs and WKYs). 0 W: newborn rat; 2 W: 2-week-old rat; 4 W: 4-week-old rat; 6 W: 6-week-old rat; and 8 W: 8-week-old rat. Magnification: 400X. **b** Masson’s trichrome revealed a significant expansion of the interstitial space in kidney cortices from 8-week-old SHRs compared to the cortical areas of WKY kidneys (SHR vs. WKY; *p < 0.05). Results are mean ± SEM; *n* = 5

Figure 1a shows, from week 4 of life, the degree of tubulointerstitial fibrosis in the renal cortices of SHR kidneys. Compared to those, WKY kidneys showed a lower collagen accumulation in the expanded interstitium along with cellular interstitial infiltrates in the cortex. In addition, SHR kidneys at week 8 had more significant interstitial collagen deposition compared to what was seen in WKY kidneys. The interstitial fibrotic area in SHRs revealed a twofold expansion of the interstitial space compared to WKYs (50 ± 8 vs. 25 ± 10 %; *p* < 0.05, *n* = 5) (Fig. 1a).

**Fig. 2:**
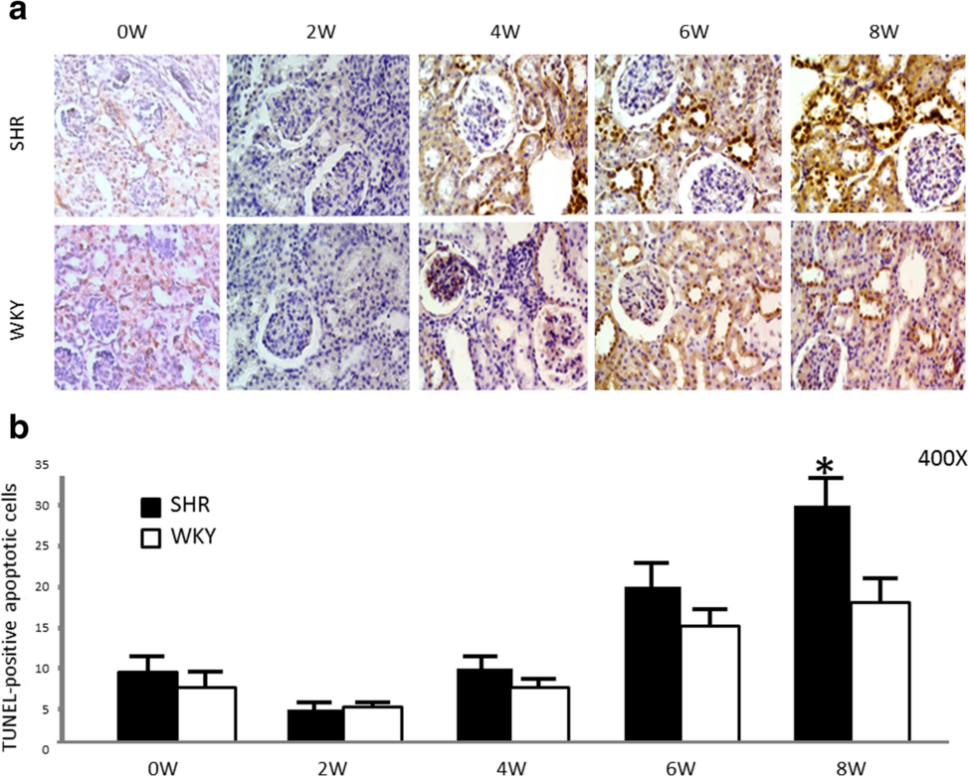
Identification and quantification of cellular apoptosis. **a** Localization of apoptotic nuclei by TdT-uridine-nick-end-labeling technique. Apoptotic nuclei appear as heavy brown-stained nuclei in tubule epithelial cells. In 8-week-old rats, apoptotic nuclei in SHR renal cortices appear as heavy brown-stained nuclei in collecting ducts and in lesser proportions in proximal tubules, whereas there is a slight increase in apoptotic cells in the epithelium from collecting ducts and proximal tubules in WKY kidney cortices. Magnification: 400X. **b** Quantification of apoptotic epithelial cells in cortices. More TUNEL-positive nuclei were recorded in tubular epithelial cells of renal cortices from SHRs than in those from WKYs (8 week-old rats); *p < 0.05. Each bar represents mean ± SEM; *n* = 5

Figure 2a shows, from week 4 of life, an increased number of TUNEL-positive apoptotic cells in tubular epithelial cells from SHRs compared to those from WKYs. Interestingly, the number of TUNEL-positive apoptotic cells in 8-week-old SHRs was significantly higher than the number found in WKYs (30 ± 4 vs. 18 ± 4; p < 0.05, *n* = 5) (Fig. 2a).

**Fig. 3:**
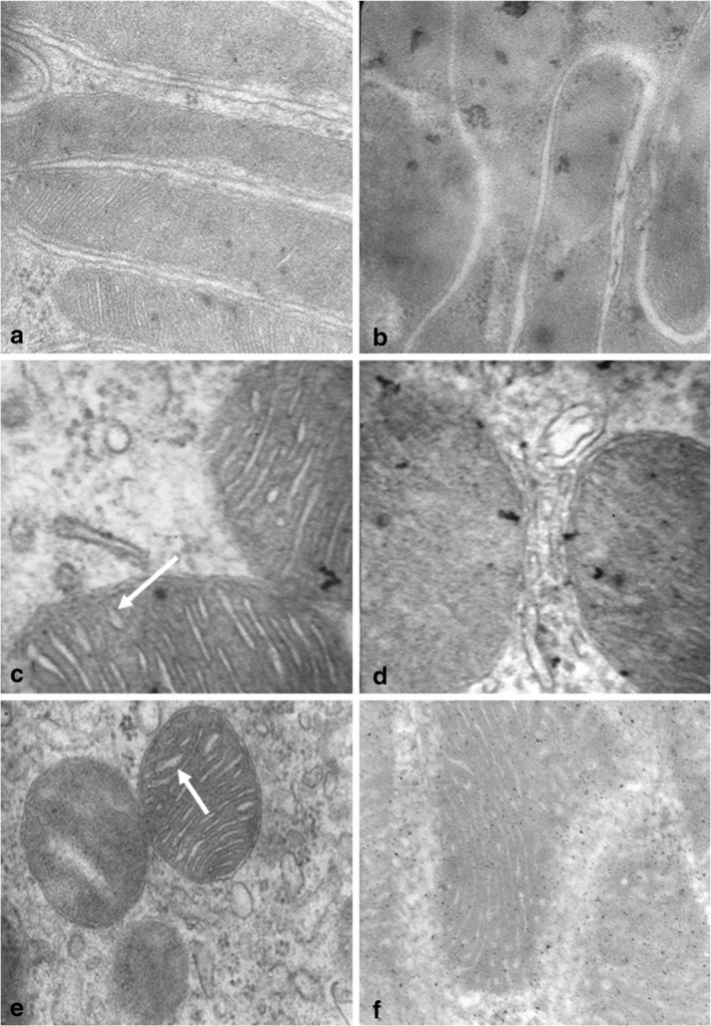
Electron microscopy study in neonatal SHRs and WKYs. Effects of hypertension development in mitochondria of renal cortical cortices of newborn SHR (**a**) and WKY (**b**), 4-week-old SHR (**c**) and WKY (**d**), and 8-week-old SHR (**e**) and WKY (**f**). Note that the mitochondria present dilated mitochondrial crestae (*arrows*; **c** and **e**) in the tubules of SHRs. Convoluted distal tubules of WKYs (**b**, **d** and **f**) showing normal ultrastructure were included for comparison. Magnification: 60,000 (**a**), 58,000 (**b**), 70,000 (**c**, **d** and **f**), and 65,000 (**e**)

Figure 3 shows the results of the electron microscopy study. In 4-week-old SHRs, the proximal convoluted tubules display normal tubular cells with baseline projections within normal parameters. They had abundant mitochondria, mostly with normal ultrastructure, except that some begin to show changes, such as wider spaces than expected between the mitochondrial cristae (luminescent spaces larger than usual, arrow). Conversely, in 4-week-old WKYs, the space between cristae was normal. On the other hand, the architecture of the renal tissue obtained from 8-week-old SHRs was composed of disorganized cortical tubules, and the cellular structure was also altered. These cells contained vacuoles of different sizes, mitochondria with cristae (arrow in SHR) separated by widened spaces between them –some of them containing vacuoles and dense bodies surrounded by membranes. In 8-week-old WKYs, the cytoplasm and mitochondria showed a normal appearance for this type of cell and, in addition, the cortical area of the renal tissue was well-preserved.

### Effects of hypertension development on glomerular morphometry

**Fig. 4:**
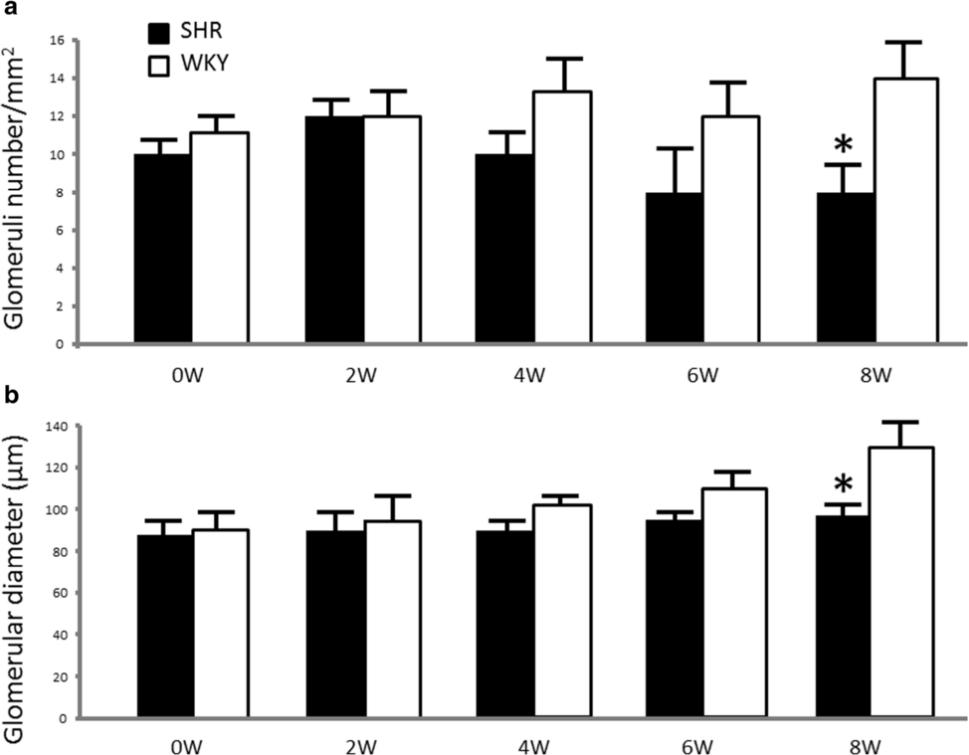
Effects on glomerular number and size during hypertension development. **a** The relative number of glomeruli per square millimeter of renal cortex was significantly lower in 8-week-old SHRs than in WKYs (*p < 0.05). No substantial changes in number were observed among WKYs during the 8 weeks studied. **b** A significant decrease in glomerular diameter was found in 8-week-old SHRs relative to WKYs (*p < 0.05). As with the number of glomeruli, no substantial changes in glomerular size were observed among WKYs. Results are mean ± SEM; *n* =5

Figure 4 shows the evaluation of glomerular morphometry. A slight decrease in the number of glomeruli was found in 4-week-old SHRs in relation to WKYs (10 ± 1.5 vs. 13.5 ± 1.5; *p* = NS). Similarly, a slight decrease in the relative glomerular diameter was found in 4-week-old SHRs in relation to WKYs (90 ± 6 vs. 101 ± 4 μm; *p* = NS). However, at week 8, a significant decrease in the number of glomeruli was found in SHRs with respect to WKYs (8 ± 1.3 vs. 14 ± 2; *p* < 0.05. Likewise, a significant decrease in the relative glomerular diameter was found in SHRs with respect to WKYs (95 ± 6 vs. 130 ± 10 μm; *p* < 0.05).

### Expression of WT-1, VDR, Hsp70 and AT_1_, and NADPH activity during hypertension development

**Fig. 5:**
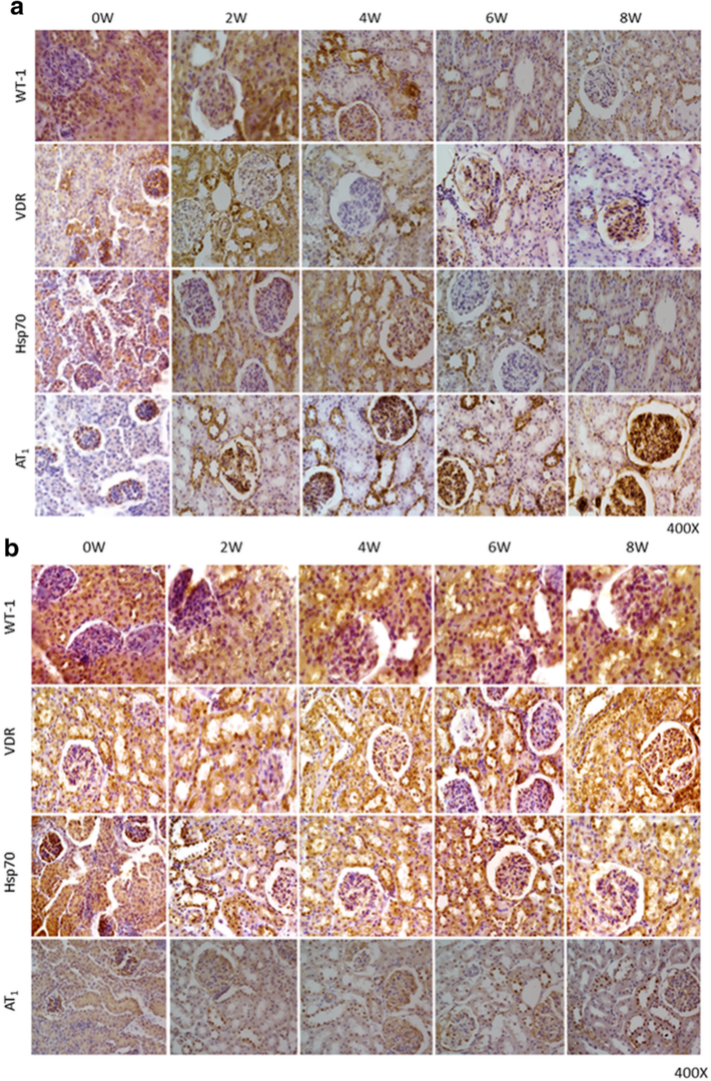
**a** Histological sections of neonatal SHR kidney cortices during hypertension development. In newborn SHRs (0 W), WT-1, VDR and Hsp70 immunostaining (expression) was relatively high, and it decreased in the cytoplasm of tubule epithelial cells and in glomeruli, while AT_1_ staining was observed in the same epithelial duct segments. After 4 weeks of hypertension evolution, decreased WT-1/VDR/Hsp70 immunostaining levels were seen in cortex tubule cells, and there was higher AT_1_ staining in tubule cells (4 W vs. 0 W). Finally, after 8 weeks of hypertension development, a greater decrease in WT-1/VDR/Hsp70 immunostaining levels were seen in cortex tubule cells, compared to what was seen in newborn SHRs (8 W vs. 0 W). Also, a very high level of AT_1_ staining in the tubule cells and glomeruli of the 8-week-old SHR was observed (8 W vs. 0 W). Magnification: 400X. **b** Histological sections of neonatal WKY kidney cortices. In newborn WKYs (0 W to 8 W), WT-1, VDR and Hsp70 immunostaining (expression) was relatively high, while an AT_1_ low staining was observed in the same epithelial duct segments. Magnification: 400X

**Fig. 6:**
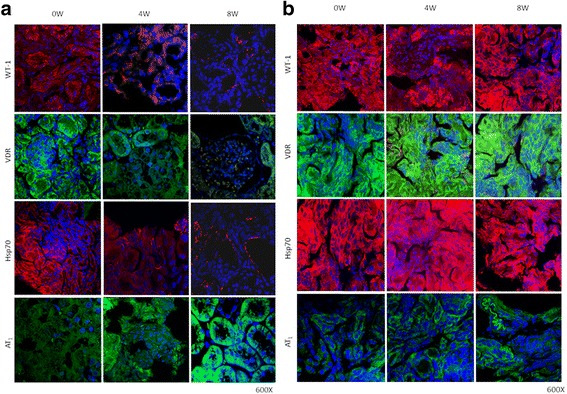
**a** Immunofluorescence confocal microscopy study in SHRs. Immunofluorescence/cytochemical localization of WT-1, VDR, Hsp70 and AT_1_ in kidney cortices from SHR rats. Renal cortex tissues were labeled with antibodies against WT-1, VDR, Hsp70 and AT_1_ followed by anti-rabbit fluorescein isothiocyanate-conjugated, and anti-mouse Rhodamine Red and Green X-conjugated secondary antibodies. Nuclei were stained with Hoechst 33342 (*blue*). Images represent five different experiments. In tubule epithelial cells of the newborn SHR (0 W), greater WT-1 (red), VDR (green) and Hsp70 (*red*) staining were identified. Similarly, basal expression of AT_1_ (*green*) appears in the tubule epithelial cells. Surprisingly, at week 4 of hypertension development, immunoreactive WT-1, VDR and Hsp70 decreased in the cytosol and the membrane of epithelial tubule cells. In addition, at week 8, these parameters were very low. Contrarily, at week 4 of hypertension development, immunoreactive AT_1_ in the cytosol and the membrane of epithelial tubule cells increased, and at week 8, it was very high. Magnification: 600X. **b** Immunofluorescence confocal microscopy study in WKYs. Immunofluorescence/cytochemical localization of WT-1, VDR, Hsp70 and AT_1_ in kidney cortices from WKY rats. Renal cortex tissues were labeled with antibodies against WT-1, VDR, Hsp70 and AT_1_ followed by anti-rabbit fluorescein isothiocyanate-conjugated, and anti-mouse Rhodamine Red and Green X-conjugated secondary antibodies. Nuclei were stained with Hoechst 33342 (*blue*). Images represent five different experiments. In tubule epithelial cells of the newborn WKY (0 W to 8 W), high WT-1 (*red*), VDR (green) and Hsp70 (*red*) staining were identified. In addition, a low expression of AT_1_ (*green*) appears in the tubule epithelial cells. Interesting, at week 8, immunoreactive WT-1, VDR and Hsp70 not decreased in the cytosol and the membrane of epithelial tubule cells. Also, at week 8, immunoreactive AT_1_ not increased in the cytosol and the membrane of epithelial tubule cells. Magnification: 600X

**Fig. 7:**
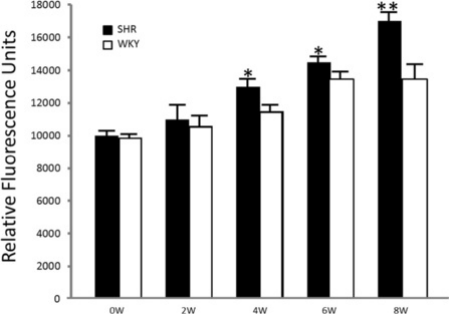
NADPH activity in mitochondrial fractions from renal cortices during hypertension development. NADPH oxidase activity was observed in SHRs and WKYs: newborn rats (0 W), 2-week-old rats (2 W), 4-week-old rats (4 W), 6-week-old rats (6 W) and 8-week-old rats (8 W). Specifically, NADPH oxidase activity in mitochondrial fractions from kidney cortices increased at week 4 and 6 of hypertension evolution (*p < 0.05, both ages), while it was considerably higher only in the 8-week-old SHR, compared to the normotensive control (WKY) (**p < 0.01). Each bar represents mean ± SEM; *n* = 5

Immunocytochemical and immunofluorescence analyses were performed to establish protein location and immunoreaction intensity. Figures 5 and 6 show the expressions of WT-1, VDR, Hsp70 and AT_1_ in the cortices of SHRs and WKYs. In the renal cortices of 4-week-old SHRs, low WT-1, VDR and Hsp70 immunostaining/immunofluorescence (Figs. 5a and 6a) was observed in the epithelial cell cytoplasm. Contrary to this, AT_1_ staining was shown in the same epithelial cells from SHRs (Figs. 5a and 6a). These values were very significant at week 8 of the study, when a very low WT-1, VDR and Hsp70 immunostaining/immunofluorescence (Figs. 5a and 6a) was observed in the epithelial cell cytoplasm. Conversely, a very high AT_1_ staining was shown in the same epithelial cells from SHRs (Figs. 5a and 6a). On the other hand, in all renal cortices (4, 6 and 8-week-old) of WKYs, high WT-1, VDR and Hsp70 immunostaining/immunofluorescence (Figs. 5b and 6b) was observed in the epithelial cell cytoplasm. Contrary to this, AT_1_ low staining was shown in the same epithelial cells from WKYs (Figs. 5b and 6b). These values were not significant. Conversely, a very low AT_1_ staining was shown in the same epithelial cells from SHRs (Figs. 5b and 6b). Furthermore, preliminary results in SHRs by western blotting and RT-PCR (*n* = 3) support these results (Additional files 1: Data S1 and 2: Data S2). Parallel to this study, Fig. 7 shows, in mitochondrial fractions from cortices at week 4, that NADPH oxidase activity was significantly greater in SHRs than in WKYs (13,000 ± 500 vs. 11,500 ± 500 RFU/μg prot/min; *p* < 0.05, *n* = 5). Interestingly, the NADPH oxidase activity in 8-week-old SHRs was significantly higher than the activity found in the WKY group (17,000 ± 600 vs. 13,500 ± 700 RFU/μg prot/min; *p* < 0.01, *n* = 5). In consequence, the NADPH oxidase activity gradually grew throughout the study.

## Discussion

This paper is the first report showing that, in the natural history of hypertension, ultrastructural mitochondrial damage occurs first, before hemodynamic clinical manifestations appear. Therefore, altered mitochondrial energy metabolism linked to master nephrogenic factors, such as WT-1, could play a central role in the essential hypertension model, and should be further investigated. The present study shows WT-1 expression in the late nephrogenic process associated with hypertension development as well as the corresponding anatomical and functional correlation. Also for the first time, previous to the increase in blood pressure, we demonstrated low expressions of WT-1, VDR and Hsp70 in kidneys from neonatal SHRs.

As widely known, hypertension is considered one of the most important public health problems in developed countries, affecting about one billion people worldwide (9.4 million deaths/year; World Health Organization 2013). Hypertension occurs as an asymptomatic disease, although, paradoxically, many changes that precede the elevation of blood pressure produce specific organic lesions, some clinically defined. In addition, the multitude of contributing factors (environment, sodium intake, renin, insulin resistance, sleep apnea and age, among others) makes hypertension complex to study mainly due to the genetic component associated with its appearance. Of particular interest to this study is the relationship between hypertension and kidney disease, which yet remains unclear and is a matter of considerable research interest, with evidence suggesting that hypertension is both a cause and a consequence of kidney disease.

The kidney is an extremely complex structure, consisting of functional units having about 10,000 cells divided into at least 14 different cell types. This implies that the renal morphogenesis must be perfectly orchestrated, and that the conversion of mesenchymal mesodermal cells into polarized epithelial cells [26] is a critical step of this process. There is evidence that WT-1 may be directly involved in the regulation of cell proliferation and differentiation. In situ hybridization studies have shown WT-1 to be selectively expressed in the metanephric blastema and glomerular epithelium during embryonic and fetal development [27]. The expression patterns of WT-1 indicate an important role of this gene not only during urogenital development but also during fetal and postnatal life. Furthermore, using laser microscopy techniques, WT-1 was sub-located in the nucleus cell, regardless of cell type and stage of development. Moreover, it has been concluded that the initial growth of the ureteric bud is dependent on a signal from the metanephric blastema and WT-1 would be necessary for this [28–30]. Therefore, WT-1 is essential for normal kidney development [31].

In contrast, nephrogenic deficiency has been recognized as a powerful risk factor for kidney disease, conditioning a more rapid progression of the disease and the possible development of hypertension [2]. Our group has confirmed this in the present study when quantifying/qualifying the glomeruli of SHRs compared to those of WKYs, from birth to hypertension development. Specifically, a slight decrease in the number and diameter of glomeruli was found in 4-week-old rats, while a significant decrease was found in 8-week-old rats. Similarly, we established significant changes in renal function (urea and creatinine) only in 8-week-old rats. In parallel, we could establish a close relationship of these parameters and WT-1 expression, which was clearly lowered especially in 8-week-old rats. This is consistent with previous reports from our laboratory where a low expression of WT-1 in neonatal rats with kidney disease was associated with glomerular changes and reduced nephrogenic markers such as Snail, BMP-7 and E-cadherin [32]. However, the most striking finding of the present study was to establish that, first, there were changes in WT-1 expression linked to glomerular alterations (in 4-week-old rats), and then, these were followed by hemodynamic changes with elevation of blood pressure (in 6-week-old rats). To support this, genes typically involved in the process of hypertension development are also WT-1 dependent [5, 33]. Interestingly, inherited mutations in the WT-1 gene can lead to life-threatening hypertension [5]. Thus, the renin gene transcription is regulated by the WT-1 (−KTS) protein and this could explain the findings in patients with WT-1 mutations that occur with increased plasma renin and hypertension. Therefore, and of particular interest to our current discussion, one important consequence of the altered nephrogenic process is hypertension development, which further amplifies the risk of onset and progression of kidney disease [33]; this makes it controversial to analyze whether hypertension is a cause or a consequence of kidney disease.

To emphasize our findings, WT-1 plays a key role in the onset of kidney formation, the progression of kidney formation and the maintenance of normal kidney function. The WT-1 protein participates in diverse cellular processes such as proliferation, differentiation and apoptosis and, in accordance with these functions, the number of target genes and/or proteins it affects is still increasing [34]. Particularly, the cells of the glomerulus, the proximal tubules and the distal tubules all derive from the metanephric mesenchyme where WT-1 already plays an active role [35]. According to this, in addition to glomerular alteration, we also established a significant change in the morphometric evaluation of fibrosis and apoptosis in glomerulus and tubular cells. Contrary to our results, apoptosis was markedly decreased in the kidneys of neonatal SHRs compared with their normotensive controls [36]. Nevertheless, it should be noted that the cited study was conducted in newborn SHRs within the first week of life, a period when we did not find significant changes. Previously, we had demonstrated an increase on renal interstitial fibrosis and apoptosis in adult SHRs [10]. However, this is the first study using the essential hypertension model which established a lesser WT-1 expression level occurring parallel to renal morphometric changes during late renal development and its consolidation as an adult organ. This is a key finding, considering that nephrogenesis in rats continues many days after birth. Thus, renal development in the newborn rat is comparable to that of the midtrimester human fetus, while renal maturation in the 2-week-old rat is analogous to that of the human infant [37]. Interestingly, Dr. Chevalier demonstrated that renal growth is impaired and nephron number is reduced in 14- to 19-day-old rats with obstructive nephropathy [38]. Using the same model, we have reported a significantly low expression of WT-1 in neonatal rats linked to glomerular changes and reduced nephrogenic markers [32]. Hence, kidney impairment is a significant event in hypertension development and, thus, alteration of a key nephrogenic gene expression such as WT-1 appears to contribute to hypertension development.

On the other hand, recent studies in hypertension have shown low levels of vitamin D linked to RAAS exaltation [6, 39, 40]. Moreover, Hsp70 regulates signaling pathway responses to cellular oxidative stress; and, in that respect, our laboratory has shown that Hsp70 protects against angiotensin II-induced hypertension [9]. We examined the effect of losartan on the expression/localization of Hsp70 in primary cultures of proximal tubular cells of SHRs, and demonstrated that the membrane translocation of Hsp70 could exert a protective effect by reducing the activity and expression of NADPH oxidase. In line with these studies, we also demonstrated in adults SHRs that structural and functional changes were reversed by VDR induction and that the association with enalapril worked even better. Thus, our recent data suggest that a low AT_1_ expression through VDR induction could be a consequence of Hsp70-mediated cell protection [10]. These concepts could be critical in understanding the complex interplay between VDR and RAAS for hypertension and related inflammatory disorders. Unprecedentedly, and occurring previous to the increase in blood pressure, we demonstrated low expressions of VDR and Hsp70 linked to a poor WT-1 level in the renal cortices from 4-week-old SHRs. Contrary to this, AT_1_ staining was observed in the same SHR epithelial cells. These values were very significant in 8-week-old rats. These data are revealing in the light of previous contributions. WT-1 and Hsp70 are physically associated in rat kidney cells, in primary Wilms’ tumor samples and in cultured cells with inducible expression of WT-1. Hsp70 is recruited to the characteristic sub-nuclear clusters containing WT-1. In addition, WT-1 requires amino-terminal transactivation domain for binding to Hsp70, and the expression of that domain itself is sufficient to induce the expression of Hsp70. Replacing the Hsp70 binding domain is sufficient to restore the functional properties of WT-1. These observations indicate that Hsp70 is an important cofactor for the function of WT-1, and suggest an emergent role of Hsp70 during nephrogenesis [11]. VDR, a ligand-activated transcription factor, is also regulated by WT-1. In humans, multiple assays suggested that, while WT-1 can choose from three binding sites within the VDR promoter, the VDR gene activation seems to occur through a single site. This site differs from a site-sensitive WT-1 previously identified in the murine VDR promoter [12]. Accordingly, the evidence suggests that there could be a modulation between Hsp70, VDR and WT-1. Moreover, the decoupling between Hsp70 and VDR associated with a possible poor expression of WT-1 could be keys to hypertension development.

In the present study, we have demonstrated that there was ultrastructural damage at the mitochondrial level in the renal cortices of neonatal SHRs. In addition, a presence of high AT_1_ and NADPH oxidase activity coupled with low WT-1, VDR and Hsp70 expressions were consistent with the histological and structural changes demonstrated. Importantly, hypertensive-induced renal injury is characterized by activation of several deleterious pathways, including oxidative stress, RAAS, renal remodeling, poor vitamin D levels and apoptosis, all of which may compromise mitochondrial integrity and function. Mitochondria are the main generators of reactive oxygen species (ROS) in cells, yet their pathophysiological role in hypertension and related inflammatory diseases remains to be fully clarified [13]. Mitochondrial dysfunction has been implicated in the etiology of many diseases, like hypertension, and, among the many factors involved, our laboratory has characterized in mitochondria the RAAS exaltation and deficiency of vitamin D receptor [6, 10, 39]. In this paper, we have demonstrated that proximal convoluted tubules display normal tubular cells with baseline projections within normal parameters. They had abundant mitochondria, mostly with normal ultrastructure, except for some beginning to show changes, such as wider spaces than expected between the mitochondrial cristae. Moreover, in 8-week-old SRHs, the architecture of the renal tissue was disorganized, and the cellular structure was also altered. These cells contained vacuoles, mitochondria with cristae separated by widened spaces between them –some of them containing vacuoles and dense bodies surrounded by membranes. In the last decade, it has been shown that ROS and inflammation, with special attention to mitochondria, play an important role in the development of hypertension [13, 41]. In this regard, our laboratory proposed a complex interplay between inflammatory pathway regulation and Hsp70 [42]. One mechanism that has recently been discussed by our work group and other laboratories is the possible involvement of Hsp70 and WT-1 on mitochondrial signaling pathway, where the net effect would favor cell survival by WT-1 stabilizing Bcl_2_, and would limit the potential for release of cytochrome C from mitochondria [15]. Also, Hsp70 interacts with the VDR and plays a role in controlling VDR concentrations within cells [43]. Vitamin D has also been demonstrated to be a nontoxic inducer of Hsp70 in the rat kidney [44]. NADPH oxidase activity was reverted in mitochondrial fractions from vitamin D inducer-treated animals [19]. Furthermore, VDR-modulated Hsp70/AT_1_ expression may protect the structure and function of SHR kidneys [10]. In addition, transcriptional activation of the VDR by WT-1 can mediate programmed death of renal embryonic cells in response to 1,25-(OH)(2)D(3) [45]. Thus, previous evidence and our present findings support a key role of the vitamin D endocrine system in renal cell growth and differentiation during hypertension development. Moreover and in accordance with our current findings, maternal vitamin D deficiency accompanies changes in the expression of important renal factors that can slow maturation of glomeruli, extending the period of nephrogenesis [46].

**Fig. 8:**
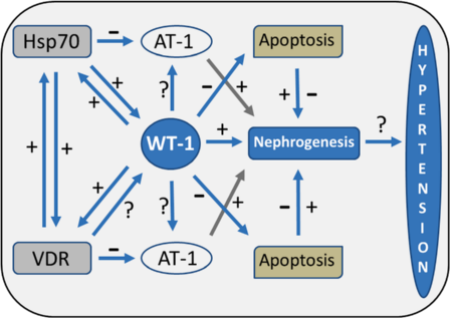
Proposal of hypothesis on mitochondrial dysfunction as hypertension precursor. The potential for interaction between Hsp70 and VDR related to mitochondrial disturbances could be a consequence of WT-1 modulation

Collectively, we propose an original hypothesis holding mitochondrial dysfunction as a precursor of hypertension. The potential for interaction between Hsp70 and VDR in mitochondria could be a cause or consequence of WT-1 modulation (Fig. 8, proposed hypothesis graph).

## Conclusions

The originality of the proposal presented in this paper is the idea that, in the natural history of hypertension, ultrastructural mitochondrial damage occurs first, before hemodynamic clinical manifestations appear. The altered mitochondrial energy metabolism linked to master nephrogenic factors, such as WT-1, could play a central role in the essential hypertension model, and should be further investigated. Unprecedentedly, we show opposing relationships between blood pressure levels in relation to the WT-1 nephrogenic factor and related genes. To our knowledge, this is the first study that finds that hypertension may be a result of possible changes in renal development by decoupling organogenic key factors. Finally, although the relationship between hypertension and kidney disease remains unclear and a matter of considerable interest for research, our current results provide new evidence suggesting that essential hypertension would result as a consequence of kidney disease with nephrogenic disturbances. Further studies should be designed to validate our findings and allow the implementation of novel therapeutic tools for hypertension.

## Availability of data and materials

The authors state that all the data supporting your findings is contained within the manuscript.

### Ethics approval

The authors ensure that the manuscript adheres to ARRIVE guidelines. Animals were cared in accordance with the Guiding Principles in the Care and Use of Animals of the United States National Institute of Health. All experimental procedures were previously approved by the Institutional Animal Care and Use Committee of the Medical Sciences College, National University of Cuyo (Protocol approval N° 46/2015).

## Additional files

Additional file 1: Data S1. RT-PCR expression in SHRs cortex kidney. Representative gel of WT-1, VDR, Hsp70 and AT_1_ mRNA expression in SHRs cortex kidney. Housekeeping gene β-actin expression is shown in the line here below. (TIF 26384 kb) Additional file 2: Data S2. Protein expression in SHRs cortex kidney. Representative blot of WT-1, VDR, Hsp70 and AT_1_ protein expression in SHRs cortex kidney. Housekeeping gene β-actin protein expression is shown in the line here below. (TIF 26384 kb)

## Abbreviations

AT_1_: angiotensin II receptor, type 1
Bcl_2_: B-cell lymphoma 2
BMP-7: morphogenetic protein 7
Hsp70: heat shock protein 70
KTS: retinoblastoma-like protein 1
NADPH: nicotinamide adenine dinucleotide phosphate-oxidase
NS: not significant
RAAS: renin-angiotensin-aldosterone system
RFU: relative fluorescence units
ROS: reactive oxidative stress
SBP: systolic blood pressure
SHR: spontaneously hypertensive rats
TUNEL: terminal deoxynucleotidyl transferase dUTP nick end labeling
VDR: vitamin D receptor
WKY: Wistar-Kyoto rats
WT-1: Wilms’ tumor transcription factor 1
