# Supplementary figures and images for: Changes in renal WT-1 expression preceding hypertension development
Source: BMC Nephrol. 2016 Mar 24;17:34. doi: 10.1186/s12882-016-0250-6 (PMC4806522; doi:10.1186/s12882-016-0250-6)

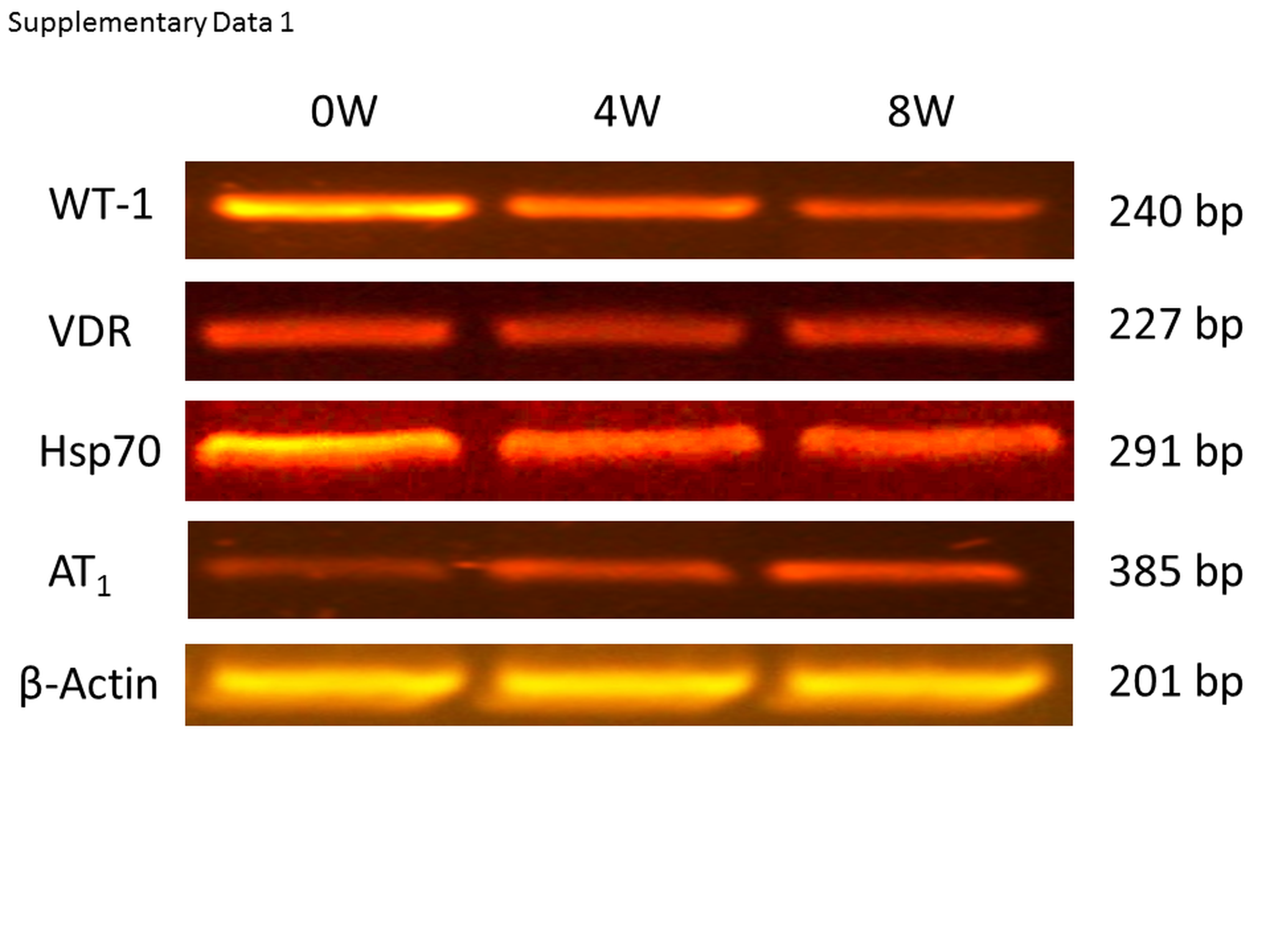

Supplement: Additional file 1: Data S1. — RT-PCR expression in SHRs cortex kidney. Representative gel of WT-1, VDR, Hsp70 and AT1 mRNA expression in SHRs cortex kidney. Housekeeping gene β-actin expression is shown in the line here below. (TIF 26384 kb) [file 12882_2016_250_MOESM1_ESM.tif]

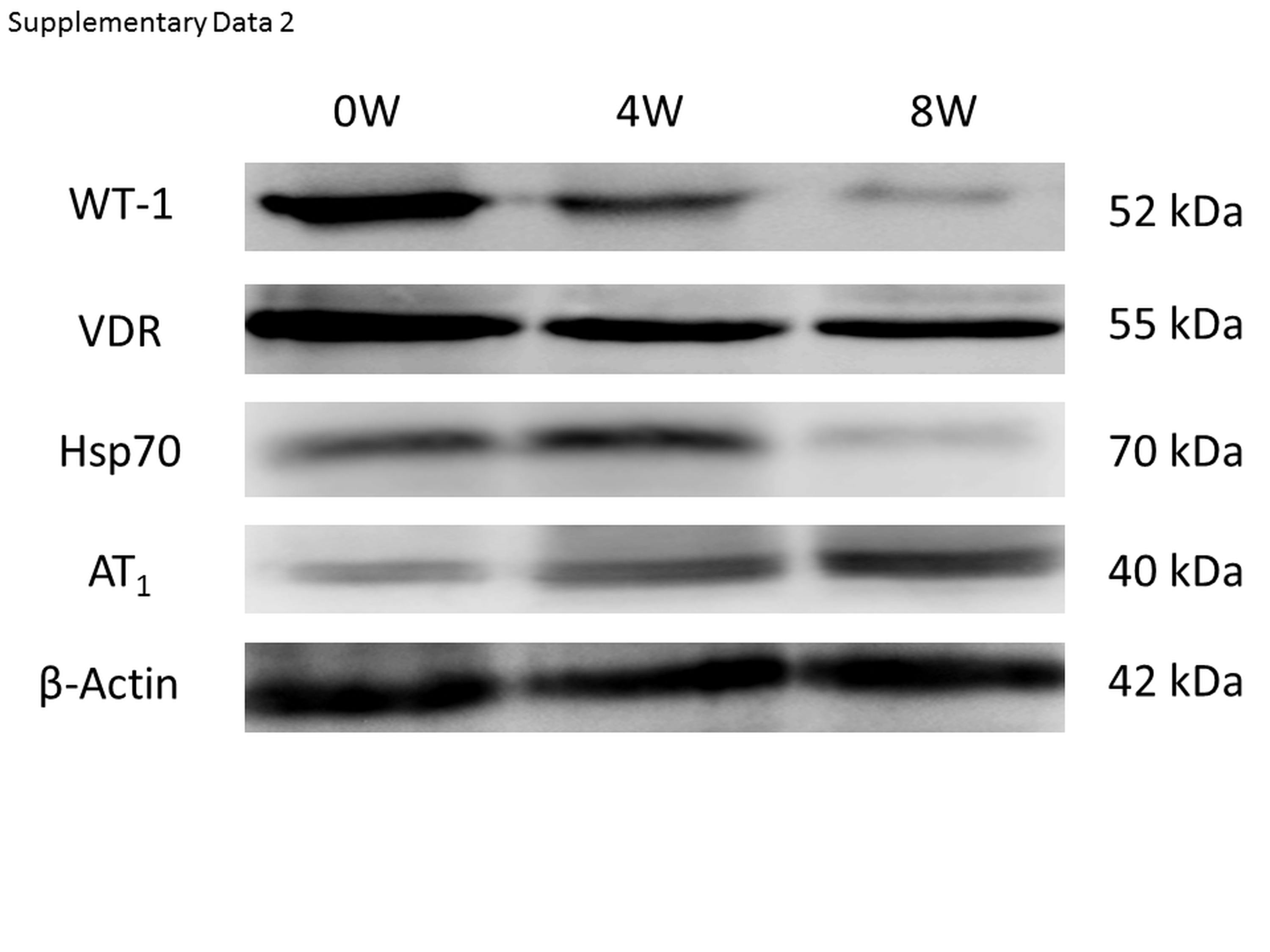

Supplement: Additional file 2: Data S2. — Protein expression in SHRs cortex kidney. Representative blot of WT-1, VDR, Hsp70 and AT1 protein expression in SHRs cortex kidney. Housekeeping gene β-actin protein expression is shown in the line here below. (TIF 26384 kb) [file 12882_2016_250_MOESM2_ESM.tif]
